# Evaluation of oxidative stress markers in ethanol users

**DOI:** 10.1590/1414-431X2023e12465

**Published:** 2023-02-27

**Authors:** L. Moraes, S.S. Dries, B.S. Seibert, R. Linden, M.S. Perassolo

**Affiliations:** 1 Programa de Residência Multiprofissional, Universidade Feevale, Novo Hamburgo, RS, Brasil; 2 Mestrado Acadêmico em Toxicologia e Análises Toxicológicas, Universidade Feevale, Novo Hamburgo, RS, Brasil; 3 Laboratório de Análises Toxicológicas, Mestrado Acadêmico em Toxicologia e Análises Toxicológicas, Universidade Feevale, Novo Hamburgo, RS, Brasil

**Keywords:** Ethanol, Oxidative stress, Antioxidant activity, Amount of alcohol, AUDIT

## Abstract

Ethanol is a central nervous system depressant that is widely consumed worldwide. When consumed chronically, it may have several consequences to the organism, such as oxidative stress. Ethanol metabolism increases the production of oxidant molecules and its consumption may cause changes in enzymatic and non-enzymatic systems that maintain cellular homeostasis. The activity of endogenous enzymes and lipid peroxidation are altered in alcohol consumers. Therefore, this study aimed to evaluate oxidative stress parameters in ethanol users compared to a control group. For that, the activity of the enzymes superoxide dismutase, catalase, and glutathione peroxidase, the ferric reducing/antioxidant power (FRAP), and malondialdehyde were evaluated. The influence of the amount of ethanol consumed on the analyzed parameters was also verified. The group of alcohol users consisted of 52 volunteers, 85% male and 15% female, with a mean age of 41±13 years. The control group consisted of 50 non-drinkers, 40% male and 60% female, with a mean age of 50±10 years. There was a significant difference in superoxide dismutase (P<0.001) and malondialdehyde (P=0.007) measurements between groups, as both parameters were increased in the group of ethanol users. Because of the higher amount of ethanol consumed, there was an increase of the catalase activity parameters and gradual reduction of FRAP. Thus, the ethanol-consuming participants were most likely under oxidative stress.

## Introduction

According to the World Health Organization (WHO), 43% of the world’s population consumed some kind of alcoholic beverage in the previous 12 months, in 2016. In addition, ethanol was responsible for approximately 3 million deaths in the same year. The deaths occurred directly from traffic accidents, drowning, and falls and indirectly from diseases such as cancer, liver cirrhosis, cardiovascular, and other digestive system diseases (1).

When consumed acutely, ethanol potentiates the action of gamma-aminobutyric acid (GABA), an inhibitory neurotransmitter (NT). It also inhibits the activity of glutamate, an excitatory NT, on the N-methyl-D-aspartate (NMDA) receptors. As a result, ethanol causes central nervous system (CNS) depression, as it increases the amount of the main inhibitory NT and decreases the main excitatory NT. Furthermore, chronic consumption leads to changes in the structure of these NT receptors, also altering their pharmacological profile (2).

Initially, alcohol causes a stimulating effect and the consumer feels more relaxed, followed by dizziness, muscle relaxation, and reduced reasoning ability. High blood alcohol concentrations can lead to CNS depression and instability of vital signs, which can lead to death (2).

**Figure 1:**
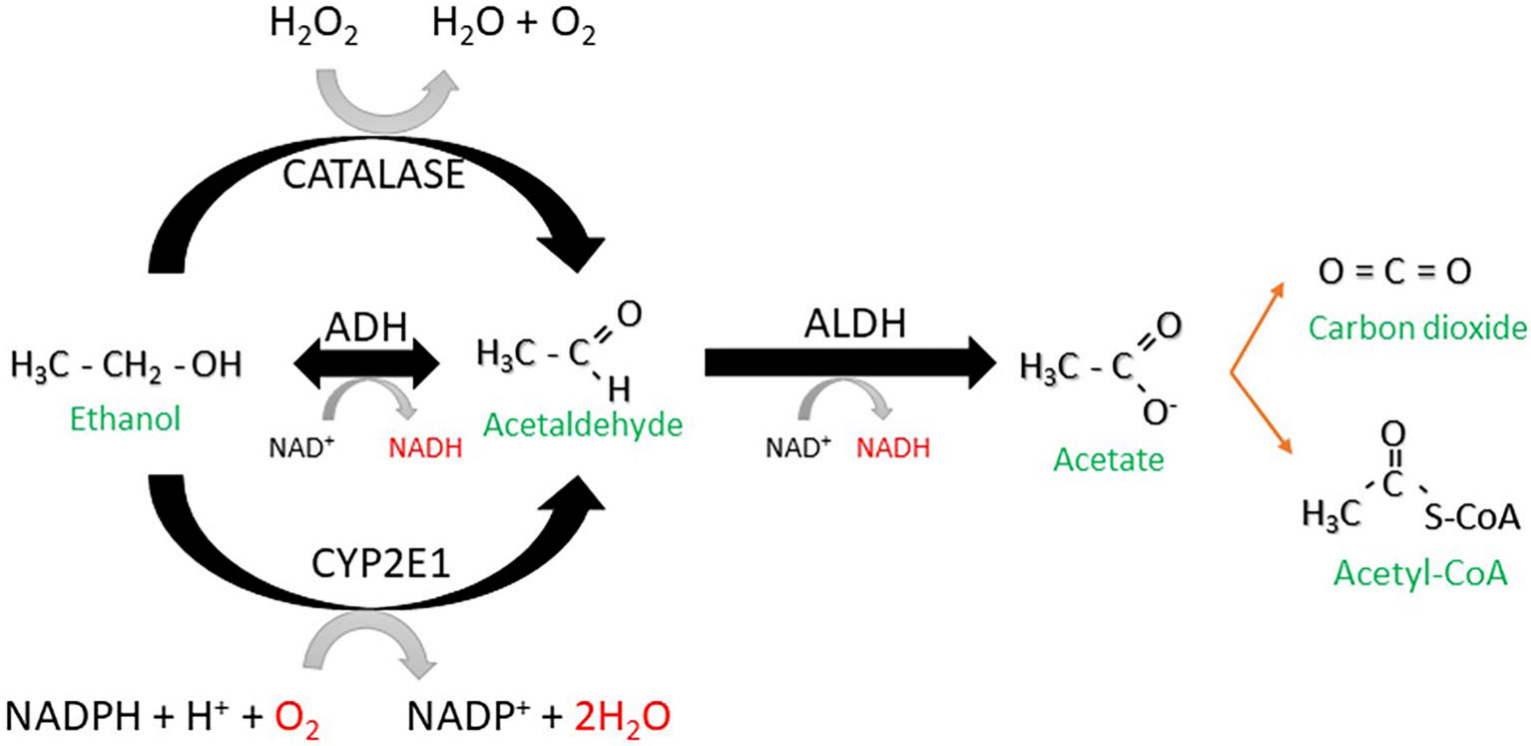
Oxidative metabolism of ethanol. The metabolism of ethanol can be carried out by four enzymes: ADH (alcohol dehydrogenase), CYP2E1 (cytochrome P450, family 2, subfamily E, member 1), ADLH (aldehyde dehydrogenase), and CAT (catalase), all of which have acetaldehyde as product.

Ethanol metabolism (Figure 1) may be carried out by three distinct enzymes: alcohol dehydrogenase (ADH), cytochrome P450, family 2, subfamily E, member 1 (CYP2E1), and catalase (CAT), all of which have acetaldehyde as a product (2). The main route is through ADH, which changes the ratio NADH/NAD+ (reduced and oxidized nicotinamide adenine dinucleotide) (2). Therefore, the mitochondria are under stress since there are more electrons entering the respiratory chain than there are oxygen molecules to receive them. Thus, more reactive oxygen species (ROS) such as H_2_O_2_ (hydrogen peroxide) and O_2_^-^ (superoxide ion) are formed (3,4).

When consumed chronically, ethanol increases the expression of the CYP2E1 enzyme, enhancing the biotransformation of ethanol through this route. However, this reaction also produces ROS such as O_2_^-^ and OH^-^ (hydroxyl radical). Catalase is more important in the brain and uses H_2_O_2_ in the reaction (2).

The formed acetaldehyde is converted into acetate by aldehyde dehydrogenase (ALDH), which may enter the bloodstream and be transformed into CO_2_ (carbon dioxide) in the heart, skeletal muscle, or brain or enter the cytoplasm and become acetyl coenzyme A (acetyl-CoA) (2).

These oxidant molecules are also generated in normal oxidative metabolism and are important for the defense against microorganisms and as second messengers. However, high concentrations may cause damage to organelles, nucleic acids, lipids, and proteins. In order to avoid such occurrence, the organism has an antioxidant defense system, which is divided into enzymatic and non-enzymatic. The former is formed by the enzymes superoxide dismutase (SOD), CAT, and glutathione peroxidase (GPx) and the latter by glutathione, vitamins A, C, and E (5), and minerals such as zinc, copper, selenium, and manganese (6).

There is no specialized enzymatic defense for OH^-^, the molecule with the highest reactive potential (6). It is formed through the Fenton or Harber-Weiss reaction in which, with the presence of iron, H_2_O_2_ and O_2_^-^, may be converted to OH^-^ (4). Due to its instability, OH^-^ may react with any nearby structure, for instance by removing a hydrogen from a polyunsaturated fatty acid, thus initiating the process of lipid peroxidation and altering the function of the biological membranes. In order to preserve biological integrity, CAT and GPx enzymes are of great importance as they catalyze reactions that prevent the accumulation of H_2_O_2_ and hence prevent the formation of OH^-^ (6).

When there is an imbalance between the defense system and the production of free radicals, a process known as oxidative stress (OS) occurs. This process can result from excessive generation of ROS or reduction in the rate of removal of oxidative molecules (6). OS may lead to the development of several pathologies such as cardiovascular diseases, cancer, neurological disorders, diabetes, ischemia (7), and alcoholic liver disease, which includes hepatic steatosis, hepatitis, cirrhosis, and hepatocellular carcinoma (5).

In order to assess OS, metabolites from biomolecule oxidation may be quantified or the antioxidant capacity may be measured. One of the metabolites, for instance, is malondialdehyde (MDA), a product of fatty acids oxidation. The ferric reducing/antioxidant power (FRAP) - the iron reducing/antioxidant power - assay measures the antioxidant capacity of the plasma as a method to evaluate the antioxidant power. In addition, GPx, SOD, and CAT enzymes may be quantified (6).

The role of OS in alcohol consumption has been reported by others (4,8-15). Therefore, this study aimed to compare oxidative stress markers (MDA, FRAP, GPx, SOD, and CAT) in ethanol users with a control group. The existence of a relationship between the amount of alcohol consumed and oxidative stress was also verified.

## Material and Methods

A cross-sectional study was carried out to assess oxidative stress in ethanol users. The group of ethanol users consisted of 52 patients who were admitted to a hospital to treat alcohol dependence. The control group consisted of 50 people paired to ethanol users according to sex and age. Inclusion criteria was 18 years old or older, acceptance to take part in the study, and absence of hepatic or renal disease (without clinical symptoms of hepatic or renal injury and normal laboratory tests for these functions, data not shown). The study was approved by the Ethics Committee on Human Research of Feevale University under the number 81406617.6.0000.5348.

Patients who accepted to participate in the study signed an informed consent form. Blood samples were collected at admission to the hospital to determine OS parameters (MDA, SOD, CAT, GPx, and FRAP). The medical record was used as a source of clinical information: blood pressure, weight, height, sex, and level of education. The Alcohol Use Disorder Identification Test (AUDIT) was also applied to assess the amount and frequency of ethanol consumption. The sum of 8 points or more in AUDIT indicates excessive intake of alcoholic beverage with high risks for human health (16).

### Analysis of OS biomarkers

Blood samples were collected in tubes containing ethylenediaminetetraacetic acid (EDTA) and heparin and then centrifuged at 2800 *g* and 20°C for 10 min. Red blood cells were used for catalase quantification and plasma was sorted into aliquots, placed in plastic microtubes, identified, and stored at -80°C until analysis of other OS parameters. The plasma from EDTA-containing tubes was used in the SOD, MDA, and GPx measurements and plasma from heparin-containing tubes was used for FRAP determination. The catalase activity was determined in red blood cells (from heparin-containing tube).

### Superoxide dismutase

To determine SOD activity, the Fluka 19160 test kit (Germany) was used, which is based on the indirect method of nitrotetrazolium blue chloride (NBT). This assay uses xanthine and xanthine oxidase to generate superoxide radicals, which react with 2-(4-)-3-(4-nitrophenol)-5-phenyl tetrazolium chloride to produce a compound, which absorbs light at 450 nm. The inhibition of chromogen production is proportional to the SOD activity present in the sample. The reading was carried out using a spectrophotometer and the results are reported in U/L.

### Malondialdehyde

MDA was determined using high-performance liquid chromatography with diode-array detection (HPLC-DAD) (17). Samples were prepared using alkaline hydrolysis of 200 µL of plasma with 1.5 M NaOH in a dry bath incubator at 60°C for 30 min to release the protein-bound fraction, followed by protein precipitation with 15% HClO_4_. The mixture was centrifuged at 4°C for 10 min at 6500 *g*. Then, 25 µL of the 2,4-dinitrophenyl-hydrazine (DNPH) solution was added to 250 µL of the supernatant and the mixture was incubated for 30 min at room temperature and under light protection. An aliquot of 50 µL of the derivatization mixture was injected into the Class VP liquid chromatography system (Shimadzu, Japan). The chromatographic separation was performed in a Lichrospher Merck RP-18 ec (250×4 mm, d.i. 5 µm) column (Germany). The mobile phase consisted of 0.2% (w/v) acetic acid:acetonitrile (62:38, v/v) with a flow rate of 1 mL/min and monitoring at 310 nm.

### Catalase

CAT was quantified according to the method described by Aebi (18). The tubes containing blood (heparin) were centrifuged at 2800 *g* and 20°C for 10 min and plasma and leukocytes were discarded. After that, red blood cells were washed 3 times with a 0.9% NaCl solution. A 1-mL aliquot of red blood cells was transferred to another tube to which 4 mL of water (dilute solution 1) was also added. Phosphate buffer solution (9980 µL) pH 7.0 was added to 20 µL of the dilute solution 1 (dilute solution 2). The reading in a spectrophotometer was carried out at a wavelength of 240 nm at 0 and 15 s. A blank was prepared for each sample. Blank was composed by 0.5 mL buffer + 1 mL of dilute solution 2 and the sample of 0.5 mL of a 30 mM (millimolar) hydrogen peroxide solution + 1 mL of dilute solution 2. Results were recorded in seconds and corrected for patient hemoglobin.

### Glutathione peroxidase

The determination of GPx enzymatic activity was carried out through the method described by Pleban et al. (19). First, a working solution was prepared with 50 mmol/L of Tris buffer pH 7.6 containing 1 mmol of Na_2_ EDTA, 2 mmol of reduced glutathione, 0.2 mmol of NADPH, 4 mmol of sodium azide, and 1000 U of glutathione reductase. The mixture was incubated for 5 min at 37°C. In order to determine the enzymatic activity of plasma, 50 µL of undiluted plasma was added to 950 µL of the working solution. Activity of GPx was recorded in U/L of plasma. After 30 s, the decrease in absorbance will be linear with time. The reaction was initiated with the addition of 10 µL of H_2_O_2_ (8.8 mmol/L), which was followed by a decrease in NADPH that was observed for 3 min at a wavelength of 340 nm. Blank was composed by water instead of plasma.

### FRAP

FRAP was determined by the method described by Benzie et al. (20), which is based on the reducing potential of the ferric ion. Blood plasma was mixed with FRAP (10 mM 2,4,6-tripyridyl-s-triazine (TPTZ) in 40 mM hydrochloric acid, 300 mM acetate buffer pH 3.6; 20 mM FeCl_3_^.^6H_2_O) which, when in low pH and in the presence of antioxidants, is reduced and forms an intense blue color that was monitored by measuring the change in absorbance at 593 nm. The change in absorbance is directly related to the combination of the “total” reducing power of electron-donating antioxidants, which are present in the reaction mixture. FRAP was calculated using ascorbic acid and a ferrous sulfate solution as standard.

### Statistical analysis

All data were stored in a database using the SPSS 24.0 software (IBM, USA). Normality of the variables was tested by the Shapiro-Wilk test. Comparisons between groups were analyzed by Student's *t*-test or Mann Whitney U test (nonparametric variables) and by chi-squared test for categorical variables. The relationship between variables was assessed through Spearman's correlation. Results were considered statistically significant when P<0.05.

## Results

One hundred and two patients took part in the study, 50 in the control group and 52 ethanol users. Most participants of the ethanol group were male (85%) and did not complete elementary school (42%), mean age was 41.1±12.6 years, BMI (body mass index) was 23.76±3.67 kg/m^2^, systolic blood pressure (SBP) was 122.5±18.8 mmHg, and diastolic blood pressure (DBP) was 77.5±13.1 mmHg. The control group was mostly composed of females (60%), 40% did not complete elementary school, mean age was 50.8±9.9 years, BMI was 23.73±2.06 kg/m^2^, SBP was 116.3±21.3 mmHg, and DBP was 76.8±15.5 mmHg. There was a significant difference in sex (P<0.001) and age (P<0.001) between the two groups. The sociodemographic characteristics are shown on Table 1.

**Table 1:** Sociodemographic characteristics of users and nonusers of alcohol.

	Control	Alcohol	Total	P
Gender				
Male	20 (40%)	44 (85%)	64 (63%)	<0.001*
Female	30 (60%)	8 (15%)	38 (37%)	
Total	50 (100%)	52 (100%)	102 (100%)	-
Education				
Incomplete Elementary School	20 (40%)	22 (42%)	42 (41%)	0.505
Complete Elementary School	8 (16%)	12 (23%)	20 (19%)	
Incomplete High School	7 (14%)	9 (17%)	16 (16%)	
Complete High School	10 (20%)	6 (12%)	16 (16%)	
Incomplete Higher Education	2 (4%)	3 (6%)	5 (5%)	
Complete Higher Education	3 (6%)	0	3 (3%)	
Total	50 (100%)	52 (100%)	102 (100%)	-
Age (years)	50.8±9.9	41.1±12.6	-	<0.001*
BMI (kg/m^2^)	23.73±2.06	23.76±3.67	-	0.966
SBP (mmHg)	116.3±21.3	122.5±18.8	-	0.120
DBP (mmHg)	76.8±15.5	77.5±13.1	-	0.801

BMI: body mass index; SBP: systolic blood pressure; DBP: diastolic blood pressure. Data are reported as absolute values/percentage (education and gender) and means±SD. *P<0.05, *t*-test and chi-squared test.

The results of OS markers of the control and ethanol group are reported in Table 2. There was a significant difference in SOD (P<0.001) and MDA (P=0.007) parameters. SOD activity was higher in ethanol users compared with the control group, 433 U/L (267-1200) *vs* 93.9 U/L (82.24-397.92), respectively. MDA, a marker of lipid peroxidation, was also higher in ethanol users, 1.47 µM (1.14-1.95) *vs* 1.25 µM (1.06-1.5), respectively. CAT, GPx, and FRAP did not present significant differences.

**Table 2:** Markers of oxidative stress in users and nonusers of alcohol.

Enzymes	Control (n=50)	Alcohol (n=52)	P
SOD (U/L)	93.9 (82.24-397.92)	433 (267-1200)	<0.001*
CAT (K/s)	1.67 (0.38-3.84)	0.95 (0.21-3.38)	0.137
GPx (U/L)	23.07 (4.85-53.24)	6.9 (2.4-63.5)	0.164
FRAP (µM)	1143.14 (810.25-2762.38)	1344 (1200-1691.6)	0.325
MDA (µM)	1.25 (1.06-1.5)	1.47 (1.14-1.95)	0.007*

SOD: superoxide dismutase; CAT: catalase; GPx: glutathione peroxidase; FRAP: ferric reducing/antioxidant power; MDA: malondialdehyde. Data are reported as median, 25th, and 75th percentiles. *P<0.05, Mann Whitney U test.

To evaluate the effect of the amount of alcohol consumed on OS markers, the AUDIT median was calculated. Thus, ethanol users were divided into two groups: AUDIT lower and higher than 24.5 (Table 3). Considering that the higher the AUDIT, the greater the amount of alcohol consumed, catalase activity was higher in people who ingested a higher amount of alcohol, from 0.56 K/s (0.07-2.17) to 1.12 K/s (0.27-4.47) (P=0.048). Other markers did not present significant differences.

**Table 3:** Oxidative stress markers in low (AUDIT <24.5) and high (AUDIT ≥24.5) ethanol users.

Enzymes	Ethanol users AUDIT <24.5 (n=26)	Ethanol users AUDIT ≥24.5 (n=26)	P
SOD (U/L)	533 (267-1550)	417 (305-988)	0.960
CAT (K/s)	0.56 (0.07-2.17)	1.12 (0.27-4.47)	0.048*
GPx (U/L)	12.34 (3.27-101.92)	4.07 (2.17-25.44)	0.272
FRAP (µM)	1515 (1207-1902)	1292 (1184-1484)	0.083
MDA (µM)	1.81 (1.24-1.99)	1.37 (1.09-1.81)	0.165

AUDIT: Alcohol Use Disorder Identification Test; SOD: superoxide dismutase; CAT: catalase; GPx: glutathione peroxidase; FRAP: ferric reducing/antioxidant power; MDA: malondialdehyde. Data are reported as median, 25th, and 75th percentiles. *P<0.05, Mann Whitney U test.

The correlation between OS markers and AUDIT was also assessed. In the present study, the only significant result was the negative correlation between FRAP and amount of alcohol consumed, that is, the higher the alcohol consumption (higher AUDIT) the lower the FRAP (Table 4).

**Table 4:** Correlation between oxidative stress markers and AUDIT.

OS markers	AUDIT	
	Spearman's correlation	P
SOD (U/L)	0.033	0.852
CAT (K/s)	0.167	0.236
GPx (U/L)	-0.188	0.182
FRAP (µM)	-0.299	0.033*
MDA (µM)	0.065	0.654

OS: oxidative stress; AUDIT: Alcohol Use Disorder Identification Test; SOD: superoxide dismutase; CAT: catalase; GPx: glutathione peroxidase; FRAP: ferric reducing/antioxidant power; MDA: malondialdehyde. *P<0.05, Spearman's correlation.

## Discussion

MDA and SOD concentrations were higher in the group that chronically consumed ethanol compared to the control group. Regarding MDA, the literature shows similar results (8,9,21): alcohol drinkers have a higher concentration than the control group, indicating damage from oxidative stress. On the other hand, controversial results are found for enzymes responsible for eliminating ROS.

MDA is a stable molecule that is more membrane-permeable than other ROS and is the final product of the degradation of polyunsaturated fatty acids. It can react with proteins and DNA (deoxyribonucleic acid) to form adducts that are linked to several pathological states, for instance: Alzheimer's disease, Parkinson's disease, cardiovascular and liver diseases, diabetes, and cancer (22). Studies that used the TBARS method to determinate MDA activity also reported higher levels in ethanol users (9-12,23,24). Other studies also reported increased MDA activity, but the difference was not significant (25-27). When MDA was evaluated in red blood cells, again higher results were found in ethanol users (21).

Research has shown that a detoxification period of two weeks positively impacted MDA activity, which was reduced (8,23) and was not different from the control in some individuals (10,11). However, Wu et al. (27) and Dries et al. (28) did not find a difference in this biomarker before and after dependence treatment.

The SOD enzyme is responsible for the conversion of O_2_^-^ into H_2_O_2_ (6), and in the present study it had a higher plasma concentration in the alcohol group than in the control group. This may be explained by a compensatory mechanism to eliminate possible O_2_^-^ excess. Previous studies had similar results (8), with decreased concentrations in ethanol users (10,11,24) and with no difference between groups (27,29).

Studies that assessed SOD erythrocyte activity also reported conflicting results: higher activity in the alcohol group (21,30), lower values in alcoholics (12,23), and no significant difference (25,31).

In some studies, detoxification did not cause a significant alteration in SOD concentrations (10,11,27,28). Others found decreased concentration compared to values at hospital admission (8,29).

In addition to helping in ethanol metabolism, the CAT enzyme is responsible for removing H_2_O_2_ by generating O_2_ and H_2_O, thus avoiding the formation of OH^-^ (6). There was no significant difference of CAT in red blood cells in the present study and in other studies (21,23,29,31). This may be explained by the fact that CAT is mainly found in the brain and it was analyzed in red blood cells. However, some studies found higher CAT activity in the control group (8,12,30). Studies that analyzed the enzyme activity in serum did not find significant differences (10,11). When the enzyme activity was evaluated in alcoholic liver patients, different results were found: increased concentration in the control group (13,26,32,33), increased concentration in the alcohol group (34), and no difference (35).

In some studies with a detoxification period of two weeks, the concentration of CAT decreased compared to the control group and at the time of hospital admission (10,11). Others found no difference between the two time points (8,23,27-29).

The GPx enzyme also removes H_2_O_2_ and generates H_2_O (6). The present study did not find significant differences in plasma concentration between the two groups, which was similar to the result of another study (29). Some studies found a higher concentration of the enzyme in the control group (8,11,24) and some in the alcohol group (27). Some studies evaluated the activity of the enzyme in red blood cells and found increased levels in the control group (23) and no difference between groups (8,21,25,30,31). Studies with people with alcoholic liver disease found either higher concentrations in ethanol users (32) or no significant difference compared to control (26,35).

GPx concentration was unchanged after detoxification in some studies (8,10,11). Other studies found an increase (28) and others a decrease of activity (27,29) after two weeks of treatment.

Another way to assess the antioxidant capacity is by the FRAP method. The lower the FRAP, the greater the amount of free iron that can catalyze OH^-^ formation (6). There was no significant difference between alcohol users and controls, but an inverse correlation was found between amount of alcohol consumed and FRAP. Therefore, alcohol negatively impacts the antioxidant power, reducing defenses and increasing the potential for damage by oxidative stress. A study found a higher FRAP concentration after treatment for alcohol dependence, indicating an improvement of the antioxidant defense after detoxification (28).

The amount of alcohol consumed directly impacted only CAT, which may be explained by the fact that this enzyme is involved in the metabolism of ethanol. In addition, this enzyme is responsible for the elimination of H_2_O_2_, the product of SOD. Therefore, an increase in SOD activity could lead to an increase in CAT. A study was carried out to compare markers of oxidative stress in people who consume small or moderate amounts of alcohol and chronic alcoholics. The SOD and GPx enzymes had higher concentrations in alcoholics compared to the group of mild ethanol consumers. The concentration of MDA was higher in chronic alcoholics and moderate consumers compared to people who ingested a small amount of alcohol (36).

A study in rats was carried out to evaluate whether the amount of alcohol impacted markers of oxidative stress. There was a progressive decrease in SOD activity according to the increase in dose. There were no significant alterations in CAT, GPx, and MDA (37).

Due to the difficulty in finding a control group that matched the alcohol group, the variables sex and age were significantly different, which could affect the outcome of the study. For this reason, the influence of the variables on OS markers was analyzed. No correlation was found regarding age. However, an influence on SOD values was observed for sex - levels were higher in males [367 (320-1521) U/L] compared to females [99 (114-710) U/L].

The different results found in the literature may be due to the effect of higher concentrations of ROS on enzymes. Free radicals may inhibit the activity of enzymes or the concentration of enzymes may increase to eliminate the excess of oxidative molecules. Both mechanisms indicate oxidative stress and are associated with an abundancy of MDA. Furthermore, markers of oxidative stress may be influenced by diet, physical exercise (6), and diseases such as diabetes (38), asthma and other respiratory diseases (39), hypertension, and dyslipidemia (40).

The increased formation of O_2_^-^ because of ethanol metabolism leads to increased SOD activity to prevent the accumulation of this radical. Therefore, more H_2_O_2_ is generated, enabling an increase of OH^-^, which is the molecule that initiates lipid peroxidation, increasing MDA. Therefore, the ethanol drinkers in this study were under oxidative stress, which is evidenced by the higher concentration of these two markers.
